# 16p11.2 microdeletion syndrome: a case report

**DOI:** 10.1186/s13256-018-1587-1

**Published:** 2018-04-03

**Authors:** D. Dell’Edera, C. Dilucca, A. Allegretti, F. Simone, M. G. Lupo, C. Liccese, R. Davanzo

**Affiliations:** 1 Unit of Cytogenetic and Molecular Genetics, “Madonna delle Grazie” Hospital, 75100 Matera, Italy; 2 Unit of Neonatology and Pediatrics, “Madonna delle Grazie” Hospital, Matera, Italy; 3 Department of Pharmaceutical Sciences, University of Padua, Padua, Italy

**Keywords:** Developmental delay, Intellectual disability, CGH-array, Submicroscopic chromosomal changes, 16p11.2 microdeletion syndrome

## Abstract

**Background:**

The recurrent ∼ 600 kb 16p11.2 microdeletion is among the most commonly known genetic etiologies of autism spectrum disorder, overweightness, and related neurodevelopmental disorders.

**Case presentation:**

Our patient is a 2-year-old white girl from the first pregnancy of a non-consanguineous healthy young white couple (father 33-years old and mother 29-years old). Our patient and her parents’ DNA were analyzed by comparative genomic hybridization-array platform. Comparative genomic hybridization-array analysis highlighted a ∼ 600 kb deletion in 16p11.2 region. It has a segregant nature, since it was found in the mother and in her 2-year-old daughter. The microdeletion was confirmed by fluorescence *in situ* hybridization analysis.

**Conclusions:**

The presented clinical case is worthy of note since the observed microdeletion is often associated with a clinical phenotype tending to overweightness, but the proband (female) was hospitalized due to poor height and weight development, and anorexia. Moreover, the segregant nature of the observed genomic abnormality has to be noted, as well as the phenotypic variability between the mother and daughter. The case described here enriches the phenotypical spectrum linked to the 16p11.2 microdeletion. For these reasons, in the presence of a suspected genetic pathology it is fundamental to study the proband from the clinical point of view, to extend the clinical observation to the parents, and to provide a good family anamnesis. In this way, it is possible to reveal the presence of a familial genetic pathology whose phenotypical outcomes can be highly variable among the members of a family.

## Background

**Fig. 1:**
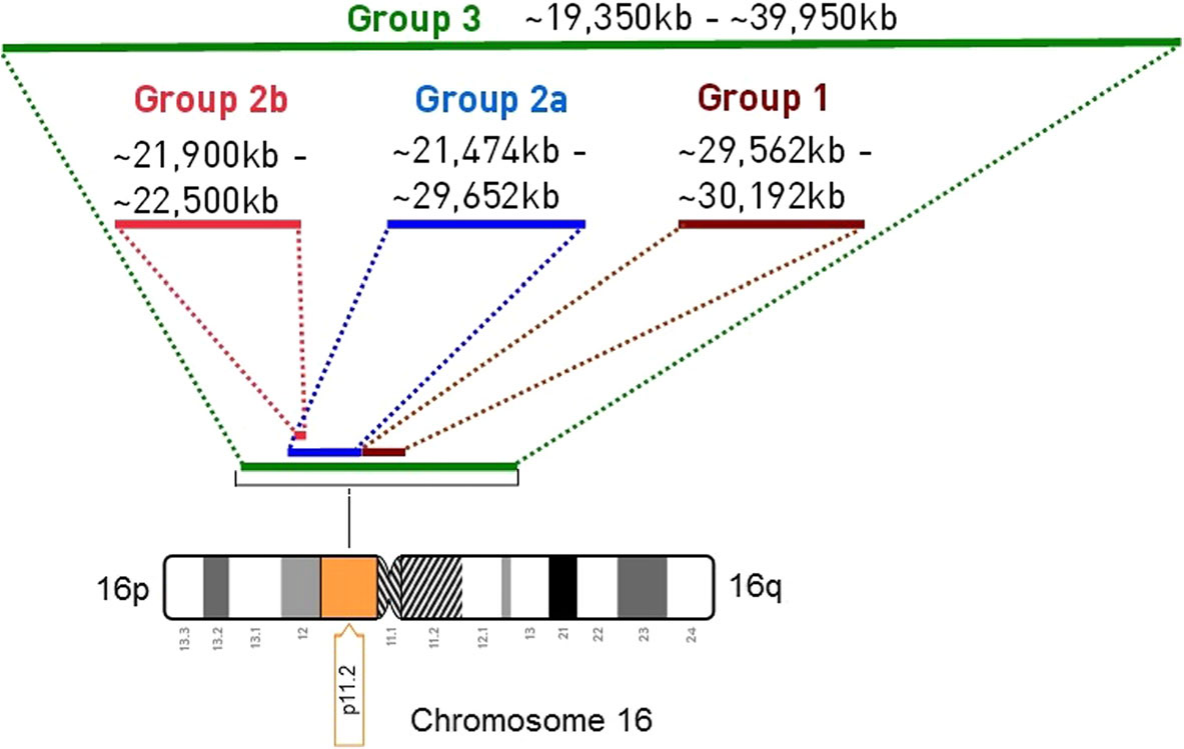
The 16p11.2 microdeletion is grouped into three groups (explanation in the text)

16p11.2 microdeletion syndrome, Online Mendelian Inheritance in Man (OMIM) #611913, is a rare genetic disorder. There are different categories, or designations, used to describe 16p11.2 deletions based on the location and amount of genetic material deleted. In general, people with a 16p11.2 microdeletion belong to one of three groups (Fig. 1):

- **Group 1** Typical microdeletion of a ~ 600 kb region containing 29 genes [1]. This deletion has a population prevalence of approximately 1/2000 [2] and reaches 0.5% in autism spectrum disorders (ASD) [3–7]. It is one of the most commonly known single locus etiologies of neurodevelopmental disorders and ASD [8]. We and others have demonstrated that this deletion predisposes to a highly penetrant form of obesity with a 43-fold increased risk of developing morbid obesity [9].
- **Group 2a/2b** has deletions that do not overlap with Group 1 and are closer to the end of chromosome 16; this is called the “distal” 16p11.2 region.
- **Group 3** has larger deletions that encompass all of the genetic material missing in group 1 and group 2.

This deletion can happen in a couple of different ways. It can be de novo, meaning that the deletion is brand new in the family. Most often, 16p11.2 deletions are de novo; various studies have found that close to 75% of children (three out of four) with a 16p11.2 deletion did not inherit it from their mother or father. However, in some families, the deletion is inherited; meaning that either the mother or the father also has the 16p11.2 deletion and has passed it to his or her children. If a parent of a child with a 16p11.2 deletion is found to have the deletion as well, there is a probability of 50% that his or her other children will have the same deletion.

The 16p11.2 microdeletion has been found in nearly 1:100 people with autism, in nearly 1:1000 people with a language or psychiatric disorder, and in nearly 3:10,000 people in the general population [10, 11].

Since a 16p11.2 deletion can be passed down from parents to their children, other family members can be examined to see if they carry the same deletion. If identified subjects with deletion 16p11.2, for preventive purposes, it is useful to propose prenatal diagnosis.

The phenotypic spectrum associated with the 16p11.2 microdeletion includes ASD, mild mental retardation (MR)/developmental delay (DD) and/or possibly other primary psychiatric disorders. The microdeletions are more likely to be penetrant and to be associated with nonspecific major or minor dysmorphism. There are probands with deletion-positive ASD with a less severe phenotype than siblings with deletion-negative ASD underscoring the significant phenotypic heterogeneity [12, 13].

In this work we report a case of a patient who was hypothetically diagnosed with: RAS/MAPK syndromes, Noonan syndrome, or Wolf-Hirschhorn syndrome. Comparative genomic hybridization (CGH)-array analysis has instead found a ~ 600 kb microdeletion lying on the short arm of chromosome 16p11.2. This genomic condition is associated with the “16p11.2 microdeletion syndrome.”

The presented case is worthy of note because the observed microdeletion is almost always associated with a clinical phenotype tending to overweightness [14–16], but the proband has been hospitalized because of poor height and weight development, and anorexia. Moreover, both the segregating behavior of the genomic abnormality and the phenotypical variability between the proband and her mother have to be underlined. This is the first report of this kind in the literature.

## Case presentation

Our patient is a 2-year-old white girl born at 39th week of pregnancy through caesarean section to non-consanguineous Italian parents. The proband’s mother is obese and suffers from mild MR and minor dysmorphism.

Her growth parameters at birth were in the normal ranges (weight 3.3 kg and length 46 cm). Apgar scores were 6/8/9. The newborn presented dysmorphic signs, which is the reason why genetic screening was performed when she was 18-months old; the genetic screening enabled us to hypothesize three pathologies: RASopathy (that is, pathologies caused by mutations on genes codifying for RAS proteins), Noonan syndrome, and Wolf-Hirschhorn syndrome. At the age of 2 years she was hospitalized at Unit of Neonatology and Pediatrics of Matera, because of poor height and weight development, and anorexia. After medical examination, the girl appeared slightly dehydrated, presented a weight of 5,910 kg and an height of 67 cm, a cranial circumference of 47 cm.

Clinical observation revealed the following: brachycephalic face with a prominent forehead and frontal bossing, slight midface hypoplasia, hypertelorism (interpupillary distance of 2.9 cm); with mildly downslanting palpebral fissures, synophrys, small nose with anteverted nostrils and deep-set nasal root, mild prognathism, deep-set posterior rotated ears, full cheeks, and prominent philtrum. She held her mouth mostly opened with a cupid bowed upper lip, full lower lip, and a slightly protruding tongue.

To better explicate the causes underlying the above described health state, the following instrumental investigations were performed:

- **Brain ultrasound**: the structures of the median line are on the axis. Normal ventricular morphovolumetry. Modest non-homogeneous of the cerebral parenchyma most pronounced in the periventricular region. Hyperechogenicity of streaked thalamus arteries.
- **Renal ultrasound**: left kidney renal pielectasia or pyelectasis with “ballooned” aspect of pelvis as of suspected joint pathy. Notes: useful scintigraphic evaluation.
- **Scintigraphy**: left calico-pyelic stasis, a little responsive to diuretic and orthostasis. Conserved parenchymal functionality but asymmetric, leading to a reduction in left kidney functionality.
- **Rachis magnetic resonance imaging (MRI)**: reported fusion of D4 to D5 and D9 to D10 vertebral bodies. Accentuation of proximal kyphosis with a tendency to reverse dorsal distal portion. Cervical hypolordosis. Conserved posterior metameric alignment.
- **Doppler color echocardiography**: normal atrium-ventricular connection of large vessels. Normal morphovolumetry of cardiac chambers. Patent foramen ovale. Slight pericardial effusion. Discrete thinning of the interventricular septum.

Parameters from routine blood tests and biochemical screenings for metabolic disorders were in the expected ranges. The karyotypes performed for the proband and her parents were normal. Before proceeding with biomolecular investigations as suggested by the geneticist, the pediatrician requested a genomic study by means of CGH array in order to exclude an eventual genomic pathology.

**Fig. 2:**
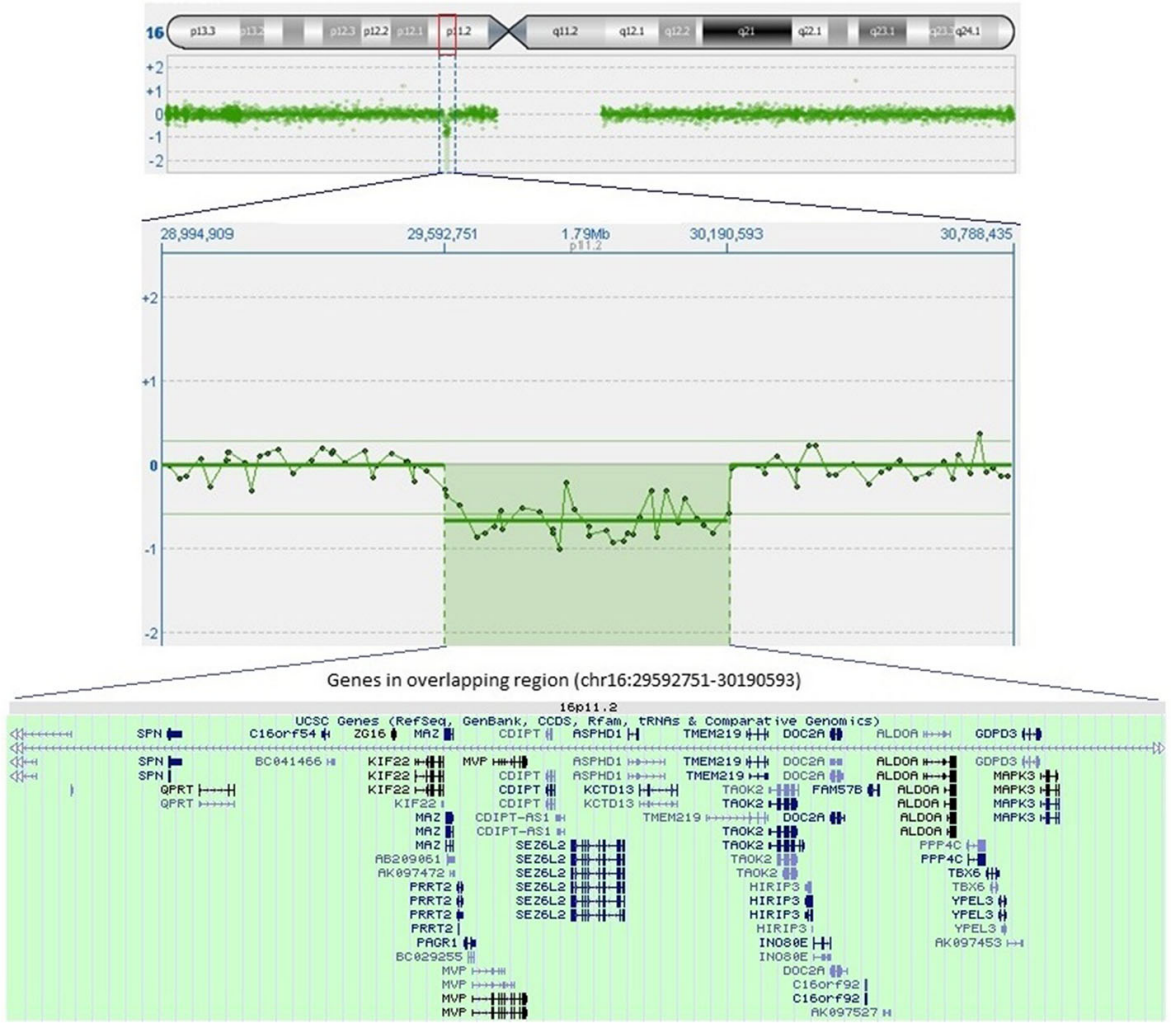
Chromosome 16p11.2 deletion in our patient. The *top panel* shows the ideogram of chromosome 16 with the 16p11.2 (29592751-30,190,593) deleted region marked in a *small red box*. The scatter plot of the array-comparative genomic hybridization data, in the *central panel*, shows a 597.84 kb microdeletion of 16p11.2 in our patient. The University of California, Santa Cruz (GRCh37/hg19 assembly) genes in the overlapping region are shown in the *bottom panel*

A CGH array highlighted a 597,84 microdeletion within the short arm of chromosome 16 (16p11.2). Based on the dimension of the deleted genomic fragment, 16p11.2 microdeletion syndrome is part of the first group (Fig. 2).

The microdeleted chromosomic region (29,592,751-30,190,593) contains the following OMIM genes: **ALDOA**, CDIPT, DOC2A, FAM57B, GDPD3, HIRIP3, KCTD13, **KIF22**, MAPK3, MAZ, MVP, PAGR1, PPP4C, **PRRT2**, QPRT, SEZ6L2, SPN, TAOK2, **TBX6**, YPEL3 and ZG16.

To verify if the observed microdeletion was a de novo mutation or has been inherited from parents, a CGH array on parental samples was performed. The analysis highlighted that the proband’s mother presented the same microdeletion as the daughter.

## Methods

### Cytogenetic analysis

Peripheral blood samples obtained from the proband and her parents were cultured for 72 hours in RPMI medium, supplemented with 20% fetal calf serum and phytohemagglutinin.

Metaphase chromosomes were analyzed by standard G-banding using Wright’s stain technique. None of the karyotypes exhibited cytogenetic alterations.

### CGH array

#### DNA preparation

Genomic DNA of the blood was obtained from the proband and her parents after obtaining signed informed consent. Genomic DNA was isolated from ethylenediaminetetraacetic acid (EDTA)-K3 peripheral blood lymphocytes by using a QIAamp DNA Mini Kit (Qiagen, Germany). DNA concentration and purity were determined with a NanoPhotometer P-Class (IMPLEN, Schatzbogen, Germany).

#### CGH array

Genomic DNA of a normal female control was obtained from Promega (cod: G1521). Array-based CGH analysis was performed using commercially available oligonucleotide microarrays containing approximately 180.000 60-mer probes with an estimated average resolution of approximately 25 Kb from CytoSure ISCA v2 180 K, Oxford Gene Technology (OCT). DNA labeling was executed using the CytoSure Genomic DNA Labelling Kit (OGT, 020020). The amount of patient DNA and controls of the same sex used was 1 μg in a final volume of 18 μl. Both DNAs were mixed with 10 μl of Random primer and 10 μl of Reaction Buffer to a total volume of 38 μl. The mix was denaturated at 99 °C for 20 minutes and then incubated in ice for 5 minutes. Each sample was added to 10 μl of deoxycytidine triphosphate (dCTP) labeling mix, 1 μl of Cy5-dCTP (test sample), 1 μl of Cy3-dCTP (reference sample) and 1 μl of Klenow and the mix was incubated at 37 °C for 2 hours, at 65 °C for 10 minutes, and then in ice for 5 minutes. Labeled samples were subsequently purified using purification columns (Amicon Ultra-0.5 mL). Labeling efficiency was determined using NanoPhotometer P330 (Implen). Each patient’s dye-labeled DNA and reference DNA was combined with 5 μl of Cot Human DNA (SureSeq OGT, 500,028), 11 μl of 10X aCGH Blocking Agent, and 55 μl Agilent 2X HiRPM Hybridization Buffer (Agilent technologies, 5188–5220). These mixtures were denatured at 94 °C for 3 minutes, pre-incubated at 37 °C for 30 minutes, and hybridized to the array in a hybridization chamber (OGT, 800,030) for 22 hours at 65 °C in a rotating hybridization (Hyb) oven at 20 revolutions per minute (rpm; OGT, 800,010). Arrays were washed using Agilent Oligo CGH Wash Buffer 1 and 2 (Agilent 5188–5221 and 5188–5222), Acetonitryl (Sigma-Aldrich, 271,004-1 L), and Stabilisation and Drying Solution (Agilent, 5185–5979), according to the Wash Procedure in OGT’s protocol.

The slide was scanned using an InnoScan 710 Microarray Scanner (Innopsys) with a resolution of 3 μm. Data were extracted from the microarray image, the background subtracted, and then normalized using feature extraction software Mapix 8.1.1. These data were subsequently imported into CytoSure Interpret Software v. 4.8 (OGT-020022). The genomic copy number was defined by the analysis of the normalized log2 (Cy5/Cy3) ratio average of the CGH signal. The moving average was computed using four consecutive probes. Regions that reached a threshold > 0.3 were interpreted as a duplication, whereas thresholds ≤ 0.6 were interpreted as a deletion. Genomic region analyses were performed according to the human reference sequence build 37.

#### Copy number variations (CNVs) validation

CNVs were compared to the DECIPHER, DGV, International Standard for Cytogenomic Arrays (ISCA) consortium (https://www.iscaconsortium.org/index.php/search), and Troina Database of Human CNVs (http://gvarianti.homelinux.net/gvariantib37/index.php) and classified pathogenic, likely pathogenic, benign, likely benign, or of unknown significance, using the following criteria:

- pathogenic – anomalies mapping on genomic regions associated to known syndromes or involving known dosage-sensitive genes and large imbalances of de novo origin or inherited from a similarly affected parent;
- likely pathogenic – small alterations of de novo origin or inherited from a parent with a similar phenotype, involving genomic regions or genes whose possible association with clinical conditions has not been definitely identified, but could be supposed from the clinical databases (DECIPHER, ISCA and Troina);
- benign – polymorphic variants reported in several healthy individuals in more than one study within DGV and/or alterations detected in at least two patients with clearly distinct phenotypes of the present cohort;
- likely benign – microdeletions and microduplications reported in few controls in DGV, but defined benign or likely benign in the clinical databases (DECIPHER, ISCA, and Troina) and inherited from a normal parent;
- of unknown significance – inherited alterations not described or with discordant definitions among those databases [17].

### Fluorescence in situ hybridization (FISH) analysis

To confirm the CGH array results, FISH analysis was performed by means of specific commercial probes (Abbott Laboratories, Abbott Park, Illinois, USA; Vysis probe name, LSI FUS (Cen); Spectrum Orange Probe 275Kb). FISH confirmed the presence of 16p11.2 microdeletion both in the proband and in her mother.

## Conclusions

The case described in this case report is worthy of note because the observed microdeletion is almost always associated with a clinical phenotype tending to overweightness; however, the proband was hospitalized because she was underweight, of short stature and anorexic. Moreover, the observed genomic abnormality has a segregant nature. A phenotypical variability between mother and daughter has been found.

The case described here enriches the phenotypical spectrum linked to the 16p11.2 microdeletion. For these reasons, if a genetic pathology is suspected, it is fundamental to study the proband from the clinical point of view, to extend the clinical examination to her parents, and to provide a good family anamnesis. It is also essential that a multidisciplinary team carefully evaluates the patient.

In this way it is possible to highlight the presence of a genetic pathology with variable expressivity. 16p11.2 microdeletion is inherited in an autosomal dominant manner. The proband often has a de novo deletion; however, as in this case, the deletion can also be transmitted from a parent to a child. Interpretation of results from prenatal testing is challenging given the inherent difficulty in accurately predicting the phenotype.
